# Incremental impact upon malaria transmission of supplementing pyrethroid-impregnated long-lasting insecticidal nets with indoor residual spraying using pyrethroids or the organophosphate, pirimiphos methyl

**DOI:** 10.1186/s12936-016-1143-7

**Published:** 2016-02-18

**Authors:** Busiku Hamainza, Chadwick H. Sikaala, Hawela B. Moonga, Javan Chanda, Dingani Chinula, Mulenga Mwenda, Mulakwa Kamuliwo, Adam Bennett, Aklilu Seyoum, Gerry F. Killeen

**Affiliations:** National Malaria Control Centre, Ministry of Health, Chainama Hospital, College Grounds, off Great East road, PO Box 32509, Lusaka, Zambia; Malaria Elimination Initiative, Global Health Group, University of California, 550 16th St., San Francisco, CA 94158 USA; Vector Biology Department, Liverpool School of Tropical Medicine, Pembroke Place, Liverpool, L3 5QA UK; Environmental Health and Ecological Sciences Thematic Group, Ifakara Health Institute, PO Box 53, Ifakara, Morogoro, United Republic of Tanzania

## Abstract

**Background:**

Long-lasting, insecticidal nets (LLINs) and indoor residual spraying (IRS) are the most widely accepted and applied malaria vector control methods. However, evidence that incremental impact is achieved when they are combined remains limited and inconsistent.

**Methods:**

Fourteen population clusters of approximately 1000 residents each in Zambia’s Luangwa and Nyimba districts, which had high pre-existing usage rates (81.7 %) of pyrethroid-impregnated LLINs were quasi-randomly assigned to receive IRS with either of two pyrethroids, namely deltamethrin [Wetable granules (WG)] and lambdacyhalothrin [capsule suspension (CS)], with an emulsifiable concentrate (EC) or CS formulation of the organophosphate pirimiphos methyl (PM), or with no supplementary vector control measure. Diagnostic positivity of patients tested for malaria by community health workers in these clusters was surveyed longitudinally over pre- and post-treatment periods spanning 29 months, over which the treatments were allocated and re-allocated in advance of three sequential rainy seasons.

**Results:**

Supplementation of LLINs with PM CS offered the greatest initial level of protection against malaria in the first 3 months of application (incremental protective efficacy (IPE) [95 % confidence interval (CI)] = 0.63 [CI 0.57, 0.69], P < 0.001), followed by lambdacyhalothrin (IPE [95 % CI] = 0.31 [0.10, 0.47], P = 0.006) and PM EC (IPE, 0.23 [CI 0.15, 0.31], P < 0.001) and then by deltamethrin (IPE [95 % CI] = 0.19 [−0.01, 0.35], P = 0.064). Neither pyrethroid formulation provided protection beyond 3 months after spraying, but the protection provided by both PM formulations persisted undiminished for longer periods: 6 months for CS and 12 months for EC. The CS formulation of PM provided greater protection than the combined pyrethroid IRS formulations throughout its effective life IPE [95 % CI] = 0.79 [0.75, 0.83] over 6 months. The EC formulation of PM provided incremental protection for the first 3 months (IPE [95 % CI] = 0.23 [0.15, 0.31]) that was approximately equivalent to the two pyrethroid formulations (lambdacyhalothrin, IPE [95 % CI] = 0.31 [0.10, 0.47] and deltamethrin, IPE [95 % CI] = 0.19 [−0.01, 0.35]) but the additional protection provided by the former, apparently lasted an entire year.

**Conclusion:**

Where universal coverage targets for LLIN utilization has been achieved, supplementing LLINs with IRS using pyrethroids may reduce malaria transmission below levels achieved by LLIN use alone, even in settings where pyrethroid resistance occurs in the vector population. However, far greater reduction of transmission can be achieved under such conditions by supplementing LLINs with IRS using non-pyrethroid insecticide classes, such as organophosphates, so this is a viable approach to mitigating and managing pyrethroid resistance.

**Electronic supplementary material:**

The online version of this article (doi:10.1186/s12936-016-1143-7) contains supplementary material, which is available to authorized users.

## Background

Long-lasting insecticidal nets (LLINs) and indoor residual spraying (IRS) are the two first-choice malaria vector control methods available globally [1] because they can achieve massive community-wide impact upon malaria transmission, even at partial coverage [2]. This is possible because many of the world’s most potent vector species prefer people as a source of blood and must feed several times upon humans inside houses before they are old enough for infectious sporozoite-stage malaria parasites to have fully developed within them [3]. While IRS and LLINs decrease exposure of directly protected humans to infected vectors and vice versa, through contact irritancy or spatial repellency, most of the impact of LLINs and IRS upon human transmission exposure and parasitaemia results from community-level suppression of vector population density and infection prevalence, achieved by reducing their longevity through lethal exposure to their toxic active ingredients [4–6]. The success of these modes of action are influenced by the choice, dosage and formulation of insecticide utilized, as well as its coverage and mode of application, combined with the behavioural and physiological susceptibility of the targeted vector species [7–9].

Compared to IRS, LLINs coverage is much higher in most endemic countries [10, 11] due to their flexibility of delivery mechanism and cheaper costs of implementation [12]. Also, while most African vector populations predominantly feed indoors, at night [13], they may not rest on the walls after a blood meal or rest for a period insufficient to pick up a lethal dose of the active insecticide [14]. However, for LLINs to be fully effective they require deliberate active participation of individuals to use them consistently and appropriately, in addition to them being regularly replaced and kept in good repair [15, 16]. In contrast, IRS requires only initial consent by the community to have their houses sprayed and compliance with not painting or plastering over the sprayed walls for the expected duration of efficacy of the insecticide used. Additionally, a major advantage of IRS over LLINs is simply that the treated surfaces are rarely in direct contact with occupants of protected houses so the safety requirements for active ingredients that may be used are far less stringent and a much wider variety of active ingredients can therefore be used [16]. The evidence on the effects of combining IRS and LLINs varies, with some studies suggesting an incremental benefit of using both interventions [6, 17, 18], while others suggest that IRS adds no incremental impact relative to LLINs alone and/or vice versa [19–21], that LLINs alone have greater impact than IRS [22, 23] and others again indicate that the contrary is true [24, 25]. These diverse comparisons between IRS and LLINs are based on a variety of outcome measures which include impacts on vector densities or entomological inoculation rates, including prevalence, incidence or diagnostic positivity of parasitaemia among humans, and the relevant costs of providing such protection [19, 22, 24–26].

Currently there are four classes of insecticides approved for use in IRS formats: organochlorines, organophosphates, carbamates, and pyrethroids [27], but only the pyrethroids are considered safe enough for use in LLINs. The wide-scale deployment of pyrethroids in both LLIN and IRS formats has undoubtedly exerted considerable selection pressure upon vector populations, resulting in the rapid and widespread emergence of physiological resistance to these active ingredients, which may negatively influence the efficacy of LLINs in particular [28]. As a consequence, the World Health Organization (WHO) recommends a reduction in use of pyrethroids for IRS, particularly in areas where LLIN deployment has been scaled up to reach high coverage [29, 30]. Furthermore, IRS application of multiple insecticides from different classes, ideally with complementary modes of action and non-overlapping resistance mechanisms, in rotations or mosaics is recommended as the optimal means of insecticide resistance management in the short-to-medium term [28]. Unfortunately, the utilization of organochlorines for IRS, particularly DDT, has been discouraged and scaled down due to concerns about potentially negative environmental effects associated with their use [31]. The remaining recommended formulations of organophosphates and carbamates have not been extensively used in IRS programmes due to their comparatively high cost and relatively short residual periods of approximately 2–6 months [27], which necessitates spraying more than once in areas with protracted transmission seasons or perennial transmission. Fortunately, new formulations of the organophosphate pirimiphos methyl (PM) have been brought to market for public health use that appear to offer increased and prolonged efficacy, notably against pyrethroid-resistant vectors [32, 33].

Given the substantial additional cost of supplementing LLINs with IRS, especially with such expensive new insecticides, and the persisting controversy about whether incremental protection against malaria is accrued, it is important to directly evaluate such combinations at community-level with epidemiological primary outcomes and explanatory entomological secondary outcomes in representative malaria-endemic settings. Thus, the overall aim of the study was to evaluate the incremental impact of supplementary vector control with IRS upon malaria transmission by the widespread and highly efficient African vector *Anopheles funestus* in a study area with relatively high usage rates of pyrethroid-impregnated LLINs, using either one of two different formulations of pyrethroids, or one of two different formulations of the new PM organophosphate.

## Methods

### Study area

**Fig. 1:**
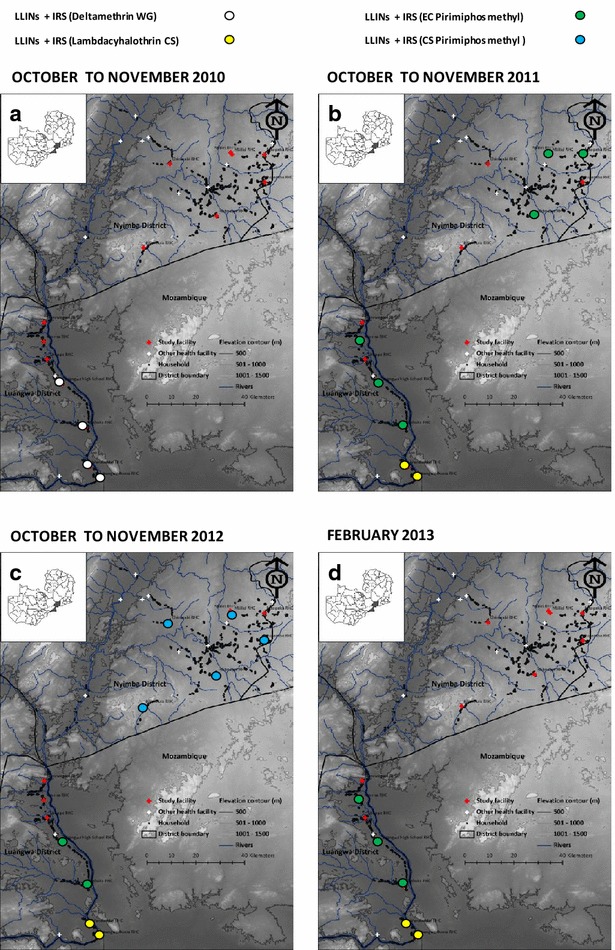
Map indicating location of health facilities and associated catchment populations enrolled in the study, with allocation of IRS treatments per cluster and year [**a** LLINs + IRS (deltamethrin WG); **b** LLINs + IRS (EC pirimiphos methyl or lambdacyhalothrin CS); **c** LLINs + IRS (EC pirimiphos methyl or CS pirimiphos methyl or lambdacyhalothrin CS); and **d** LLINs + IRS (EC pirimiphos methyl or lambdacyhalothrin CS)]

The study was conducted in the predominantly rural districts of Luangwa and Nyimba, located in Lusaka and Eastern provinces, respectively, of the Republic of Zambia (Fig. 1).

These districts have perennial transmission of *Plasmodium falciparum*, with the overwhelmingly predominant vector being *An. funestus*, which mediates a mean entomological inoculation rate (EIR) for non-users of LLINs of approximately 70 infectious bites per unprotected person per year [34]. The district of Luangwa (3468 sq km) is located 350–500 m above sea level, 325 km southeast of Lusaka, the capital city of Zambia. It has a population of approximately 27,560 residents, with an annual growth rate of 2.9 % [35]. The main economic activities in the district are fishing and agriculture. Nyimba is a larger district (10,943 sq km), with a population of 108,637 inhabitants and an annual growth rate of 3.4 % [35]. The district is located 400–1200 m above sea level, 350 km east of Lusaka. Agriculture is the predominant economic activity in Nyimba district.

### Study design

In each district, seven clusters of approximately 165 households were selected and enrolled in the study to participate in longitudinal parasite surveys [36]. Of these, 15 households in each cluster were selected and enrolled at the discretion of the community health worker (CHW), so that they were geographically distributed across the cluster, for participation in monthly entomological observations, with the exception of Luangwa High School, where 30 households were enrolled. Both parasitological and entomological assessments were conducted continuously from January 2011 to March 2013 in Luangwa and from April 2011 to March 2013 in Nyimba district in all clusters.

The pyrethroid deltamethrin [wettable granule (WG) formulation] was sprayed in all consenting households at the four southernmost clusters in Luangwa in October 2010, immediately before that year’s rainy season and initiation of this study. During the study period, three other selected IRS insecticide treatments [capsule suspension (CS) formulation of the pyrethroid lambdacyhalothrin, as well as the emulsifiable concentrate (EC) and CS formulations of the organophosphate pirimiphos methyl (PM)] were randomly allocated to clusters in advance of each rainy season. In practice this randomized allocation was not strictly adhered to by the implementation agencies in the two districts (District Medical Office (DMO) in Luangwa and Abt Associates under the supervision of the DMO in Nyimba), thus resulting in a quasi-randomized study design, described cartographically in Fig. 1. The parasitological and entomological surveys were conducted by paid CHWs as previously described [34, 36] and summarized below. In the south of Luangwa district, between October and November 2010, clusters 4, 5, 6, and 7 received pyrethroid-based IRS with deltamethrin (K-Othrine WG^®^ 250, Bayer Environmental Science, South Africa) as described in Fig. 1a. Subsequently, the organophosphate PM was introduced as an alternative insecticide for IRS in a response to detection of resistance to pyrethroids in the primary vector, *An. funestus* in Luangwa district [37–39]. The only formulation of PM that was available at the time was the relatively short-lived [33, 40] EC formulation (Actellic^®^ EC, Syngenta Crop Protection AG, Switzerland). This formulation was sprayed during the months of October and November 2011, in clusters 2, 4, 5 in Luangwa and 9, 11, 13 in Nyimba, while IRS with pyrethroid lambdacyhalothrin (Icon^®^ 10 CS formulation, Syngenta Crop Protection AG, Switzerland) was applied in only two of the four clusters in the south of Luangwa district which had been sprayed with deltamethrin the previous year, specifically in clusters 6 and 7 (Fig. 1b). The following year, in November 2012, the longer-lasting microencapsulated formulation of PM (Actellic^®^ 300CS, Syngenta Crop Protection AG, South Africa) was applied in clusters 8, 9, 10, 12, and 14, all of which were in Nyimba district (Fig. 1c). In February 2013, IRS in Luangwa district was implemented with PM-EC in clusters 2, 4 and 5 while clusters 6 and 7 received the CS formulation of lambdacyhalothrin (Fig. 1d).

### Parasitological surveys of human infection

Active monthly parasitological surveys were coupled with questionnaires recording clinical symptoms of illness, as well as access and utilization of preventive measures such as LLINs, IRS and intermittent preventive therapy (IPT), between January 2011 and May 2013, spanning a period of approximately 29 months, as described previously [36]. These surveys were conducted by paid CHWs who made active monthly visits to households that consented to participate in the study. In between active visits, study participants who developed symptoms were encouraged to seek care through passively offered diagnosis and treatment services, either from the CHWs at their place of residence or at the nearest health facility. The rapid diagnostic test used in the study was manufactured by ICT Diagnostics to detect circulating *P. falciparum* histidine-rich protein-2 antigen (ICT Malaria P.f. cassette test). All participants that were found positive for antigenaemia, which was presumed equivalent to infection, were treated with artemether-lumefantrine as per national malaria diagnosis and treatment policy [41]. In both the active and passive visits, all participants found to be negative for malaria infection but febrile or had any other complaints, were referred to the nearest health facility.

### Mosquito densities and species identification surveys

The monthly mosquito collections were conducted by paid CHWs using Centres for Disease Control and Prevention light traps (LT) and Ifakara tent traps (ITT) between January 2010 and April 2013, spanning a period of approximately 28 months, as described previously [34].

Fifteen well-distributed houses were semi-arbitrarily selected for mosquito trapping in each housing cluster, with the exception of Luangwa High School which had 30 houses due to the availability of an extra CHW involved in mosquito trapping. Each house was visited once per month for mosquito trapping using both the LT and ITT, on a consistent date of the month which was pre-agreed with each consenting household head [34]. In each consenting household, the LTs were placed at the foot end of an occupied sleeping space covered with an LLIN, hanging approximately 1.5 m above the floor. An ITT was placed immediately outside, approximately 5 m away from the house where the LT was installed and was occupied by an adult male volunteer from the same household. All the mosquito traps were set up in the evenings and collection of the captured mosquitoes was done in the early morning by aspiration. All the collected mosquitoes were initially sorted in the field to genus level by the CHWs, based on crude taxonomic features and then stored over silica until they were collected on a monthly basis and transported to a central laboratory at the National Malaria Control Centre (NMCC) for further detailed examination. Additional morphological identification of *Anopheles* to species group or complex [42] was conducted at the central laboratory of the NMCC in Lusaka. Polymerase chain reaction (PCR) for the identification of species within the *An. funestus* group [43] or *An. gambiae* complex [44] were conducted on selected samples in the NMCC laboratory.

### Vector susceptibility to different classes of insecticides

A team of trained entomological technicians from the NMCC periodically collected samples from the study sites to ascertain the susceptibility of the mosquitoes to different classes of insecticides, as background descriptive data to support appropriate interpretation of apparent impacts of various supplementary IRS treatments upon the vector population. In Luangwa district, mosquitoes were collected from cluster 2 from 2010 to 2013. However, in Nyimba district, collections were done over 3 years in different clusters (cluster 14 in 2011, cluster 9 in 2012 and cluster 13 in 2013). Adult mosquitoes were either collected while attacking humans by human landing catch (HLC) or by using pack aspirators for the indoor wall-resting mosquitoes. These were collected in cups covered with a netting material and placed in cooler box for transportation to the NMCC insectary where individual female *An. funestus* mosquitoes where allowed to feed on mouse blood so they could lay eggs that were then reared into F1 generation mosquitoes. Standard WHO susceptibility tests using insecticide-impregnated papers with discriminatory dosages of two pyrethroids (deltamethrin 0.05 % and lambdacyhalothrin 0.05 %), a carbamate (bendiocarb 0.1 %), an organophosphate (malathion 0.4 %) and an organochlorine (DDT 4 %) were carried out on 2–5 day-old F1 *An. funestus* mosquitoes. Control papers were impregnated with oil as directed by the WHO protocol [45]. Knock-down and mortality rates after 1 and 24 h post-exposure periods were recorded.

### Indoor-outdoor distribution of human exposure to *Anopheles funestus* bites

To estimate proportions of human exposure to *An. funestus* bites and malaria transmission that occurs indoors and outdoors, HLCs were conducted both indoors and outdoors by a team of trained entomological technicians from the NMCC in Lusaka and these were complemented by cross-sectional questionnaire surveys of when residents went indoors for the night, went to sleep, awoke in the morning, and left the house in the morning, as previous described [39], again as background descriptive data to support appropriate interpretation of apparent impacts of various supplementary IRS treatments upon the vector population and malaria transmission. Trained CHWs conducted HLC from 18.00 to 06.00 h, with the exception of the previously described 2010 studies where the starting time was 19.00 and finished at 07.00. The 2010 and 2011 HLC surveys were conducted in cluster 4 (Chisobe and Nyamumba villages of Luangwa district) as part of a trap effectiveness study [38], while those conducted in 2012 and 2013, where part of the quality assurance surveys were conducted in 13 clusters as part of a subsequent effectiveness assessment for a community-based trapping scheme [34]. Mosquitoes were collected for 45 min per hour to allow a 15-min break for rest and refreshment for the collectors. Each hourly collection were labelled and kept for identification to genus and species as described above. The proportion of time that residents spent outdoors and indoors, as well as asleep in bed, was estimated directly from answers to questionnaires during a cross-sectional household survey in April 2010 in Luangwa district, in which people indicated the time they usually went indoors and when they went to the bed as well as when they arose in the morning and when they left their houses [39].

### Data management and statistical analysis

The CHW Malaria Register data describing rapid diagnostic test (RDT) results associated with questionnaire responses were double entered into Excel^®^, verified, reconciled, and then cleaned following descriptive frequency analysis of the distributions of values for each variable. All entomological data were single entered, verified and cleaned prior to analysis. All statistical analyses were accomplished using SPSS version 20 (IBM) and R version 2.14.1, augmented with the lattice, Matrix and LME4 packages.

### Incremental protection of humans against malaria infection risk by IRS treatments

Previous analyses of these data collected by CHWs have demonstrated that diagnostic positivity (DP) for malaria infection, expressed as the proportion of RDT-tested individuals who were found to be positive, was a extremely powerful indicator of malaria risk that allowed numerous important epidemiological phenomena to be clearly illustrated [36]. It also proved to be a more consistent and robust indicator of geographic and temporal variation than absolute numbers of malaria infections detected, presumably because variations in CHW service utilization rates, as well as RDT and ACT availability, occur in both the nominator and denominator of DP [46], and was therefore treated as the primary epidemiological outcome used for statistical analysis of the effects of various IRS treatments, rather than incidence in terms of detected events per number of participants per unit time.

Four sequential time period categories, based on the integer number of months since the most recent spray round was completed were created for all the IRS treatments: 1–3, 4–6 and 7–12 months since beginning of the last spray round started, as well as a fifth category combining areas that had not yet received spraying during the study period and those for which the last spray round began more than 12 months ago, which was treated as the reference value. Generalized linear mixed models (GLMMs) were fitted to evaluate the association between observed malaria infection risk among human residents and the various IRS treatments applied. Malaria infection status was treated as the binary dependent, with use of an LLIN, having slept in a house that had been treated with IRS in the previous 6 months and the categorized cluster-wide IRS treatments as the independent variables of primary interest. Age category (<1, 1–4, 5–10, 11–14, 15–24, 25–44 and >45 years of age), sex, season (hot and wet from December to April, cool and dry from May to August, and hot and dry from September to November), number of previous RDTs conducted per individual and geographical location (cluster) were also included as independent variables of secondary interest (all categorical except for number of RDTs) while random effects to capture variance associated with nuisance variables of no direct interest were also included in the model (the individual identity number nested within the CHW catchment nested within the study cluster, as well as date of participant contact). IPT use was not included in the final model as an independent variable because it had no apparent effect on malaria infection prevalence (P = 0.8633). Further, in order to test for and quantify incremental impact of PM IRS as a supplement to LLINs, relative to LLINs supplemented with pyrethroid-based IRS, both pyrethroid formulations were represented by a single treatment variable, coding the same periods of months since before spraying. Similarly, in order to test for and quantify the incremental impact of the CS formulation of PM, relative to the EC formulation of the same active ingredient, as well as the two pyrethroid formulations, an additional variable was created which combined any previous treatment with any of the latter three formulations in the reference group. In all cases, incremental protective efficacy (IPE) was calculated as the complement of the odds ratio (OR) estimated directly by these GLMMs (IPE = 1−OR).

### Incremental protection of humans against human exposure to mosquito bites and malaria parasite inoculation by IRS treatments

The effect of different IRS treatment regimens on densities of *An. funestus* species were estimated by fitting GLMMs where *An. funestus* densities were treated as a dependent variable with a Poisson distribution. In order to account for variance in mosquito densities by location, identities for households were nested within those villages and then nested within clusters as random effects. Similarly, nightly temporal variance in vector density was accounted for by including date as an additional random effect. The different IRS treatment regimens were coded in terms of time period since the last round of IRS application began, exactly as described above for the epidemiological primary outcomes, so that these treatments could be included as categorical independent variables with which to detect and quantify impact upon these entomological secondary outcomes. In all, the relative rate (RR) at which mosquitoes were captured was calculated as estimated directly by these GLMMs. Unfortunately, efforts to develop laboratory capacity for determining sporozoite infection status by enzyme-linked immunosorbent assay (ELISA) at NMCC were unsuccessful so neither sporozoite prevalence nor entomological inoculation rate could be assessed as additional entomological secondary outcomes.

### Physiological resistance to insecticides

Insecticide susceptibility assays were conducted on 2–5 day-old F1 generation *An. funestus* as described by the WHO standard protocol [47] using papers impregnated with deltamethrin (0.05 %), lambdacyhalothrin (0.05 %), bendiocarb (0.1 %), malathion (0.1 %) or DDT (4 %). In order to test for time trends in physiological resistance of *An. funestus* to pyrethroids and carbamates over time, survival status of mosquitoes exposed to these insecticides in standard WHO protocols [45] was treated as the binary outcome variable in GLMMs with year as a continuous covariate and a unique identification code for each experimental replicate as a random effect. The data were stratified into sub-sets on the basis of the insecticide class, with separate models fitted for the carbamate (bendiocarb), and the combined pyrethroids (deltamethrin and lambdacyhalothrin). The model of resistance time trends for the two pyrethroids, the identities of these two insecticides within this class were included as a categorical independent variable. No such model was fitted for either the organochlorine (DDT) or the organophosphate (Malathion) because no resistance to either insecticide was apparent.

### Proportions of human exposure to *Anopheles funestus* bites occurring indoors and outdoors

The distribution of human exposure to *An. funestus* bites, and presumably malaria transmission, across different times of the night and across indoor and outdoor compartments of their living environment was calculated by weighting HLC measurements of indoor and outdoor biting rates for each hour of the night by the estimated proportion of humans indoors and outdoors during that time period, exactly as previously described [39]. These estimates of human exposure distribution across indoor and outdoor environments were calculated and presented graphically for both users and non-users of LLINs, so that the proportions of human exposure that occur indoors in the presence (*π*_*i,n*_) and absence (*π*_*i*_) of a protective LLIN could be quantified and visualized.

### Protection of human participants and ethical approval

Prior to the study, community sensitization was conducted and permission obtained from the local community leadership. Informed consent was obtained from all study participants during all surveys and spraying activities. The study team ensured that all treatment and diagnostic protocols were adhered to and that patients requiring malaria treatment received it promptly or were referred to the nearest health facility. All participants who took part in the HLCs gave written consent to participate after being informed of the risks and benefits, and were provided with weekly prophylaxis using the nationally recommended combination drug of 100 mg Dapsone and 12.5 mg pyrimethamine (Deltaprim^®^, CAPS Pharmaceuticals, Zimbabwe) so that their overall malaria risk was considered to be far lower than it would otherwise be in the course of their normal lives if they did not participate in the study [48]. All standard safety protocols for IRS application were adhered to as per national guidelines. Ethical approval was obtained from the University of Zambia, Biomedical Research Ethics Committee (Reference 004-05-09) and the Research Ethics Committee of the Liverpool School of Tropical Medicine (Approval 09.60). Authority to conduct and publish the study was also obtained from the Ministry of Health in Lusaka, Zambia.

## Results

### Characteristics of study participants and survey clusters

A total population of 25,354 people centred around health facilities in the 14 clusters participated in the study and were followed up for a period of 29 months in Luangwa and 26 months in Nyimba, starting from January 2011 and April 2011, respectively. Out of these participants, 29 % (7412) were children under the age of 5 years but DP peaked in older children between the age of five and ten. The overall cluster populations ranged from 1158 to 3429. A total of 31,974 malaria infections (21.7 % DP) were identified, which translates into an incidence of nine infections per 100 person years. The study population reported a relatively high average rate of LLIN utilization of 81.7 % of questionnaire responses over the course of the study, indicating that the respondent had slept under an LLIN the previous night, while 39.2 % of participant questionnaire responses indicated that the respondent’s house had been treated by IRS in the last 6 months. During same overall study period mean DP by cluster across all age groups and other potential stratification criteria ranged from 6.4 to 41.9 % (mean = 24.5 %), with the lowest being in the southern urban cluster and the highest in the northern rural cluster (Tables 1, 2). The potential confounding effect of LLIN ownership was excluded from the final model described in Table 2 because it had no significant effect (P = 0.7584) on diagnostic positivity.

**Table 1:** Trends in RDT-determined diagnostic positivity (DP) at each cluster over time as different IRS treatments were applied

Cluster	October 2010–March 2011		October 2011–March 2012		October 2012–March 2013	
	IRS treatment	DP % (n/N)	IRS treatment	DP % (n/N)	IRS treatment	DP % (n/N)
1	None	24.7 (372/1508)	None	9.5 (95/998)	None	14.4 (150/1039)
2	None	20.9 (559/2676)	Pirimiphosmethyl EC	8.5 (280/3292)	None	11.9 (126/1061)
3	None	26.9 (809/3006)	None	10.8 (436/4033)	None	14.1 (282/2004)
4	Deltamethrin WG	33.2 (825/2489)	Pirimiphosmethyl EC	5.9 (217/3708)	Pirimiphosmethyl EC	10.8 (314/2908)
5	Deltamethrin WG	27.5 (396/1439)	Pirimiphosmethyl EC	18.2 (624/3436)	Pirimiphosmethyl EC	27.3 (456/1673)
6	Deltamethrin WG	11.9 (338/2845)	Lambdacyhalothrin CS	5.2 (76/1457)	Lambdacyhalothrin CS	3.8 (57/1505)
7	Deltamethrin WG	6.0 (144/2415)	Lambdacyhalothrin CS	4.2 (130/3111)	Lambdacyhalothrin CS	2.99 (33/1105)
8	None	55.7 (202/363)	None	29.9 (974/3261)	Pirimiphosmethyl CS	9.0 (209/2321)
9	None	36.4 (4/11)	Pirimiphosmethyl EC	46.6 (684/1467)	Pirimiphosmethyl CS	23.5 (366/1561)
10	None	50.7 (172/339)	None	35.4 (444/1254)	Pirimiphosmethyl CS	27.1 (363/1341)
11	None	51.3 (60/117)	Pirimiphosmethyl EC	30.2 (941/3112)	None	11.9 (300/2531)
12	None	61.9 (26/42)	None	33.9 (514/1517)	Pirimiphosmethyl CS	21.7 (246/1132)
13	None	60.0 (120/200)	Pirimiphosmethyl EC	27 (5.033/1974)	None	30.6 (666/2180)
14	None	52.4 (33/63)	None	41.3 (786/1904)	Pirimiphosmethyl CS	16.99 (221/1301)

**Table 2:** Association of malaria infection status with age, sex, LLINs, IRS, number of tests conducted per participant, geographical location, season and IRS insecticide used

Category	DP %	N/N (I)	OR [95 % CI]	P
Overall	21.7	31,974/147,257 (25,354)	0.13 [0.08, 0.21]	<0.001
Age				
<1	14.2	501/3535 (1735)	1.26 [1.09, 1.45]	0.001
1–4	24.0	6127/25,505 (5677)	2.75 [2.54, 2.98]	<0.001
5–10	27.4	10,066/36,779 (7608)	3.62 [3.35, 3.91]	<0.001
11–14	26.0	4892/18,840 (4746)	3.36 [3.09, 3.65]	<0.001
15–24	20.3	4491/22,077 (5685)	2.04 [1.88, 2.22]	<0.001
25–44	14.9	4028/27,044 (5807)	1.24 [1.14, 1.34]	<0.001
≥45	13.8	1796/13,027 (2903)	1 [NA]	NA
Sex				
Male	23.3	16,068/79,208 (12,008)	1 [NA]	NA
Female	20.3	15,750/67,567 (13,228)	0.86 [0.83, 0.90]	<0.001
Interventions				
LLINs	20.0	20,613/103,149 (20,706)	0.89 [0.85, 0.93]	<0.001
IRS	17.4	7568/43,560 (9926)	0.87 [0.82, 0.93]	<0.001
Number of tests conducted per participant	21.7	31,974/147,257(25,354)	0.97 [0.97, 0.98]	<0.001
Type of visit				
Passive	43.4	6416/14,785 (8922)	1 [NA]	NA
Active	19.2	25,281/131,359 (22055)	0.29 [0.28, 0.31]	<0.001
Clusters				
*Luangwa district*				
Sinyawagora RHC	19.7	1314/6655 (1959)	2.86 [1.65, 4.97]	<0.001
Kasinsa RHC	16.7	2232/13,402 (3429)	4.67 [2.78, 7.84]	<0.001
Chitope RHC	19.6	3419/17,463 (1215)	2.92 [2.04, 4.17]	<0.001
Luangwa High School RHC	16.5	2854/17,320 (1158)	7.37 [4.31, 12.61]	<0.001
Mphuka RHC	24.9	2981/11,957 (2147)	7.37 [4.31, 12.61]	<0.001
Mandombe RHC	10.3	1386/13,508 (1805)	1.54 [0.89, 2.66]	0.119
Luangwa Boma RHC	6.4	839/13,161 (2033)	1 [NA]	NA
*Nyimba district*				
Kacholola RHC	26.7	3108/11,654 (1166)	7.50 [4.75, 11.84]	<0.001
Hofmeyer RHC	41.9	2601/6214 (2120)	15.81 [10.20, 24.52]	<0.001
Mtilizi RHC	37.6	2238/5949 (2024)	12.35 [7.72, 19.76]	<0.001
Mtilizi RHP	25.3	2478/9788 (3379)	13.49 [8.45, 21.56]	<0.001
Chinambi RHC	31.9	1740/5463 (1741)	9.16 [5.79, 14.48]	<0.001
Mkopeka RHC	32.8	2761/8413 (1311)	14.22 [8.55, 23.63]	<0.001
Chipembe RHC	32.1	2023/6310 (1916)	13.54 [8.03, 22.84]	<0.001
Season				
Hot & wet (Dec–April)	25.3	18,283/72,217 (20,243)	4.20 [3.67, 4.81]	<0.001
Cool & dry (May–Aug)	23.9	11,216/46,860 (16,513)	3.25 [2.80, 3.76]	<0.001
Hot & dry (Sept–Nov)	8.7	2444/27,983 (12,590)	1 [NA]	NA
Insecticide applied for IRS				
*Deltamethrine*				
1–3 months since last spray	13.3	322/2419 (2166)	0.81 [0.65, 1.01]	0.064
4–6 months since last spray	23.8	2411/10,150 (4231)	1.07 [0.94, 1.23]	0.295
7–12 months since last spray	13.6	2128/15,640 (4434)	1.16 [1.03, 1.30]	0.013
Never sprayed and >13 months since last spray	22.8	27,083/118,899 (23,233)	1 [NA]	NA
*Lambdacyhalothrin*				
1–3 months since last spray	4.7	145/3102 (1526)	0.69 [0.53, 0.90]	0.006
4–6 months since last spray	9.4	207/2199 (1264)	1.26 [1.01, 1.57]	0.042
7–12 months since last spray	4.5	157/3508 (1469)	0.94 [0.74, 1.21]	0.653
Never sprayed and >13 months since last spray	22.7	31,435/138,299 (24,931)	1 [NA]	NA
*Primiphosmethyl EC*				
1–3 months since last spray	18.9	1922/10,194 (5527)	0.77 [0.69, 0.85]	<0.001
4–6 months since last spray	28.8	2666/9259 (5926)	0.64 [0.58, 0.71]	<0.001
7–12 months since last spray	16.0	1793/11,184 (5760)	0.63 [0.56, 0.71]	<0.001
Never sprayed and >13 months since last spray	21.9	25,563/11,6471 (22,311)	1 [NA]	NA
*Primiphosmethyl CS*				
1–3 months since last spray	13.0	468/3590 (2675)	0.37 [0.31, 0.43]	<0.001
4–6 months since last spray	30.6	1386/4536 (3349)	0.24 [0.21, 0.27]	<0.001
7–12 months since last spray	49.5	95/192 (191)	1.35 [0.85, 2.15]	0.204
Never sprayed and >13 months since last spray	21.6	29,995/138,790 (24,588)	1 [NA]	NA

The association of malaria infection with age, sex, use of LLINs, use of IRS, geographical location (cluster), number of tests conducted per participant, season and insecticide used in IRS was determined using GLMM; with observed malaria RDT determined status as a binary dependent outcome with the independent categories of age, sex, access and use of LLINs or IRS, insecticide used in IRS, number of tested conducted per participant and seasons. The models included date and participant nested within CHW catchment nested within geographical location (cluster) as random effects except for one in which cluster was treated as a categorical variable to determine the effects of each cluster. The final model consisted of age, sex, access and use of LLINs or IRS, insecticide used in IRS, season, number of tests conducted per participant and geographical location as the determinants of malaria infection

*DP* RDT-determined diagnostic positivity, *n* Number RDT positive, *N* Total number tested by RDT, *I* Number of individuals that participated in RDT testing, *OR* Odds ratio, *CI* Confidence intervals, *P* Probability of the null hypothesis, *NA* Not applicable because reference group

**Fig. 2:**
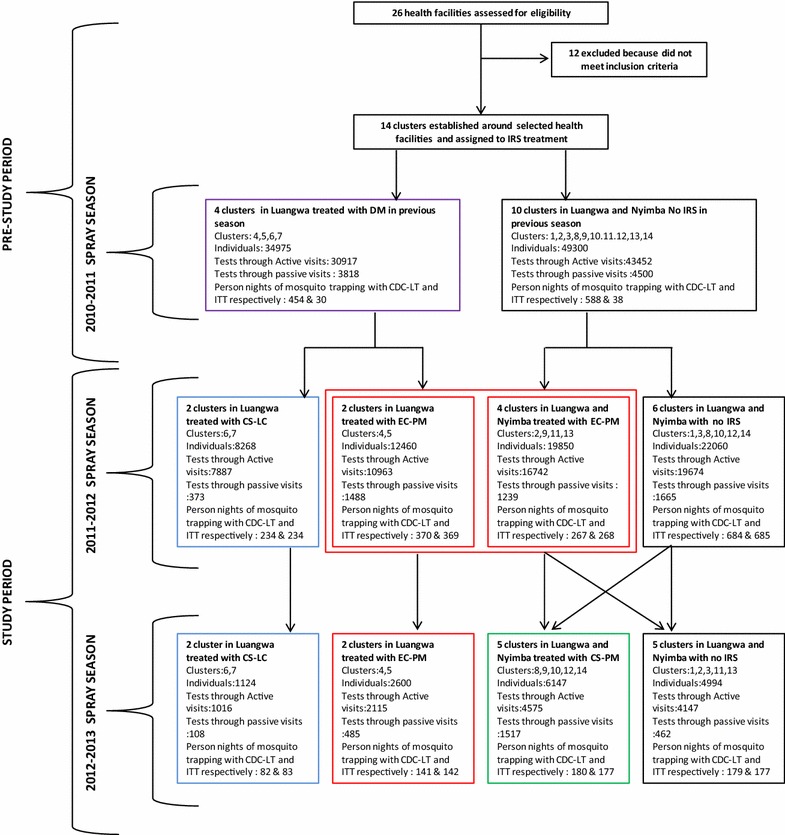
Study profile indicating treatments provided to each cluster with associated timelines, population surveyed and persons nights of mosquito trapping

The close associations of DP for *P. falciparum* malaria infection and *An. funestus* density, clinical symptoms of illness, and a variety of other factors of this setting are described in detail elsewhere based on the first year of data collection [34, 36]. The detailed profile of the study participants, and their survey contacts over the course of the entire study, are summarized in the context of the study design in Fig. 2.

**Fig. 3:**
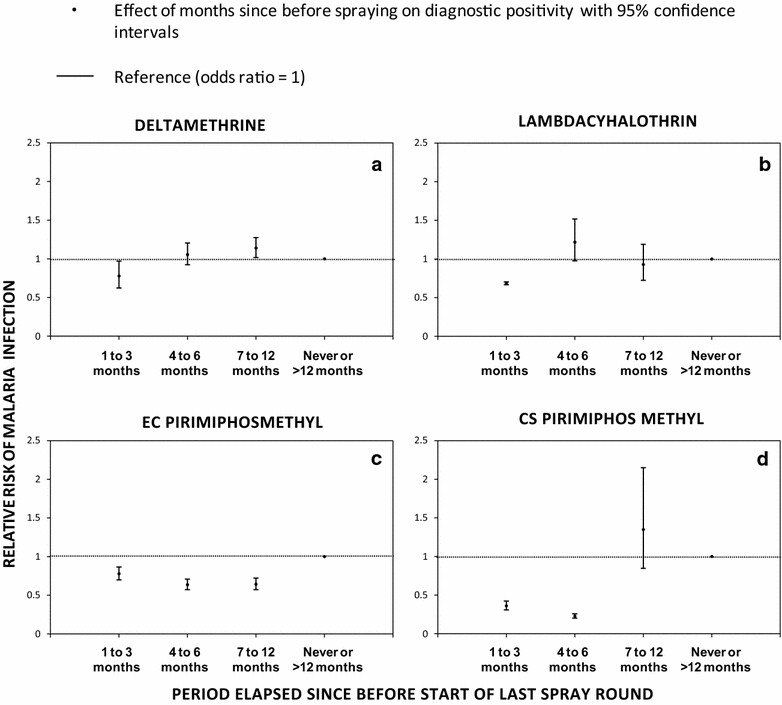
The incremental protective efficacy of each of the four IRS treatments on diagnostic positivity for *Plasmodium falciparum* malaria infection over several time periods since the last spray round began, relative to clusters that has either never been sprayed or had last been sprayed >12 months ago (reference group), estimated exactly as described in Table 1 (**a** deltamethrin, **b** lambdacyhalothrin, **c** EC pirimiphos methyl and **d** CS pirimiphos methyl)

**Fig. 4:**
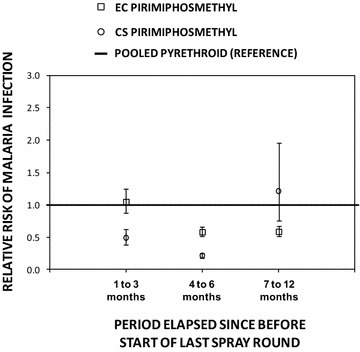
The incremental protective efficacy of pirimiphos methyl EC and CS IRS treatments on diagnostic positivity for *Plasmodium falciparum* malaria infection over several time periods since the last spray round began, relative to clusters that have been sprayed with either deltamethrin and/or lambdacyhalothrin (reference group), estimated exactly as described in Table 2, except that three separate models were fitted for the three different time periods since the last spray round began, and the combined pyrethroid formulations were treated as the reference group

**Fig. 5:**
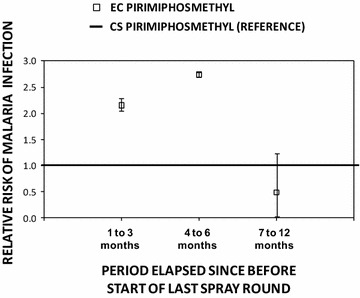
The incremental protective efficacy of pirimiphos methyl EC IRS treatment on diagnostic positivity for *Plasmodium falciparum* malaria infection over several time periods since the last spray round began, relative to clusters that have been sprayed with pirimiphos methyl EC (reference group), estimated exactly as described in Table 2, except that three separate models were fitted for the three different time periods since the last spray round began and the EC formulation of pirimiphos methyl was treated as the reference group

A descriptive comparison of summarized data restricted to the period 1–6 months post-spraying demonstrates variability among study clusters not only in IRS coverage (range = 0–100 %, mean = 29.4 %) but also LLIN use (range = 6.6–100 %, mean = 68.2 %) and diagnostic positivity (range = 2.99–61.9 %, mean = 25.4 %) (Table 1; Additional file 1). Further analysis using Pearson’s correlation, revealed a positive but weak association (r^2^ = 0.31) between IRS coverage and LLIN use, suggesting that as IRS coverage increases, so does LLIN use. However, this does not necessarily imply any causal relationship and factors which affect delivery (e.g., accessibility) and acceptance (e.g., attitudes towards malaria or mosquitoes) may well be similar for both of these vector control measures. However, there was no obvious and clear-cut effect of any particular IRS treatment in this crude descriptive comparison (Table 1; Additional file 1) so detailed regression modelling analysis was required to detect and estimate the separate impacts of these four different formulations (Table 2, Figs. 3, 4, 5).

### Magnitude and duration of incremental impact of IRS treatments as supplements to LLINs upon human risk of infection with malaria

Reported coverage of deltamethrin WG, lambdacyhalothrin CS, PM EC, and PM CS, by respondents within the first 3 months after their application in clusters to which they were assigned was 82 % (2132/2599), 61 % (2068/3384), 53 % (5909/11,078), and 69 % (2716/3913), respectively. Over the study period, DP ranged from 13.3 to 23.8 % (mean = 18.4 %), 4.5 to 22.7 % (mean = 10.3 %), 16.0 to 28.8 % (mean = 21.4 %) and 13.0 to 49.5 % (mean = 28.7 %) for clusters assigned with deltamethrin WG, lambdacyhalothrin CS, PM EC, and PM CS respectively (Tables 1, 2). As illustrated in Fig. 3, PM CS conferred the strongest initial incremental protection in the first 3 months after application (IPE [95 % CI = 0.63 [0.57, 0.69], P < 0.001), relative to LLINs alone, followed by the CS formulation of lambdacyhalothrin (IPE [95 % CI] = 0.31 [10, 47], P = 0.006), the EC formulation of PM (IPE [95 % CI] = 0.23 [0.15, 0.31], P < 0.001) and the WP formulation of deltamethrin (IPE [95 % CI] = 0.19 [−0.01, 0.35], P = 0.064). However, neither pyrethroid formulation provided any incremental protection beyond 3 months post-application, while the incremental protection provided by CS and EC formulations of PM persisted undiminished for 6 and 12 months, respectively (Fig. 3).

The first 3 months after IRS with the CS formulation of PM offered greater protection against malaria infection than IRS with pyrethroids IPE [95 % CI] = 0.51 [0.38, 0.62], P < 0.001 for LLINs + IRS with PM-CS compared to LLINs + IRS in all clusters treated with either DM-WG or LC-CS but not PM-EC (P < 0.001). The incremental protection against malaria infection by IRS with both PM formulations outlasted both pyrethroid formulations so that they both offered greater protection from 4 to 6 months post-application IPE [95 % CI] = 0.79 [0.75, 0.83], P < 0.001 for LLINs + IRS with PM-CS and IPE [95 % CI] = 0.42 [0.33, 0.48], P < 0.001 for LLINs + IRS with PM-EC, compared to LLINs + IRS with either DM-WG or LC-CS) (Fig. 4).

Beyond 6 months post-application, LLINs plus IRS with PM-CS provided no apparent incremental protection relative to LLINs alone (P = 0.204), much less LLINs + IRS with pyrethroids (P = 0.432). However, LLINs + PM-EC continued to provide incremental protection relative to not only LLINs alone (Fig. 3), but also relative to all other LLIN + IRS treatments (IPE [95 % CI] = 0.41 [0.34, 0.48], P < 0.001). When the duration of efficacy of PM-EC was examined in further detail by breaking down the third post-spray time period into two halves, it was clear that it lasted approximately a full year because similar levels of incremental protection was confirmed for both the seven and 9 months post-spray period (IPE [95 % CI] = 0.32 [0.22, 0.40], P < 0.001) and the 10 to 12 months post-spray period (IPE [95 % CI] = 0.42 [0.31, 0.52], P < 0.001).

Comparing these two IRS formulations of PM with each other as supplements to LLINs, the CS formulation confers greater protection than the EC formulation, (IPE [95 % CI] = 53.6 [0.43, 0.66]  %, P < 0.001 from 1 to 3 months post-application and 0.64 [0.57, 0.69], P < 0.001 from 4 to 6 months post-application for the contrast between LLINs + PM-CS versus the LLIN + PM-EC as the reference group) (Fig. 5). However, once the incremental benefit of supplementing LLINs with IRS using PM-CS waned after 6 months, IRS using PM-EC proved statistically superior to all other IRS formulations as supplements to LLINs for a further 6 months, including the CS formulation of the same active ingredient (IPE [95 % CI] = 0.52 [0.21, 0.70], P < 0.001 for the contrast between LLINs + PM-EC versus LLIN + PM-CS as a reference group between seven and 12 months post-application).

### Magnitude and duration of incremental impact of IRS treatments as supplements to LLINs upon human risk of exposure to bites of *Anopheles funestus*

Detailed description of the local mosquito fauna in the study area [34] showed that 34.5 % of all mosquitoes caught over the course of the study were identified morphologically as members of the *An*. *funestus* group, of which 96.5 % (575/596) of those which were successfully amplified by PCR, were confirmed to be *An. funestus*. Densities of the *An. funestus* group, as determined by routine morphological classification can therefore be considered quite reliable, as of *An. funestus,* the abundance of which is consistent with previous studies in this area [38, 39] indicating it as the overwhelmingly dominant vector of malaria in these two districts of Zambia. Therefore, subsequently in this report all mosquitoes caught from the *An. funestus* group are the nominate species in the strict sense.

**Fig. 6:**
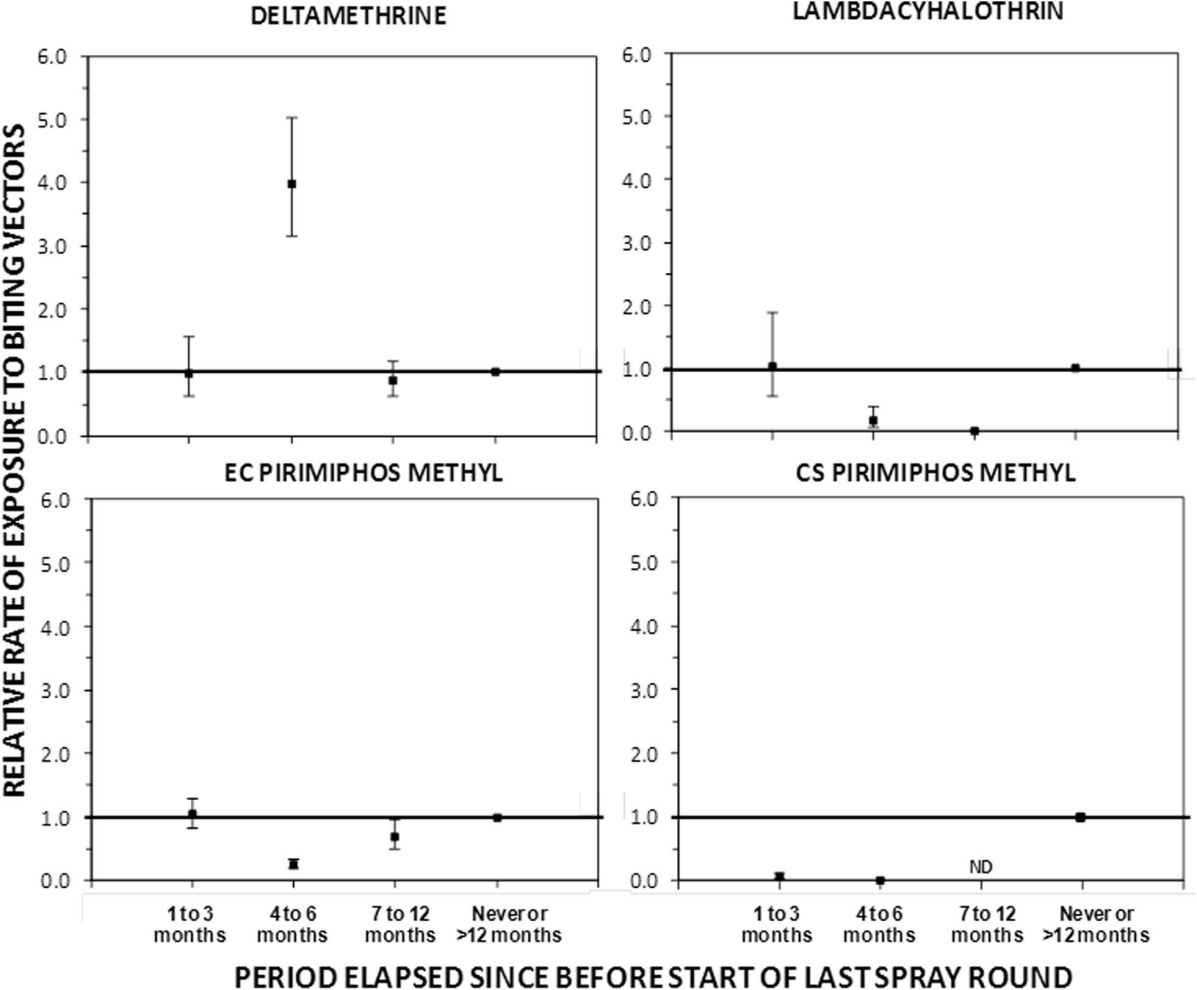
The incremental protective efficacy of each of the four IRS treatments against *Anopheles funestus* bites over several time periods since the last spray round began, relative to clusters that has either never been sprayed or had last been sprayed >12 months ago (reference group), estimated exactly as described in Table 3 (ND Not done)

The relative rates and the mean catches of *An. funestus* per IRS treatment are presented in Table 3. Relative to the times and places that had never been sprayed, or sprayed or had been sprayed >12 months previously, there were no obvious differences in the densities of *An. funestus* during the first 3 months post-spraying for both pyrethroid formulations (DM-WG (IPE [95 % CI] = 0.01 [−0.56, 0.37], P = 0.103) and LC-CS (IPE [95 % CI] = −0.03 [−0.88, 0.44], P = 0.195) and PM-EC (IPE [95 % CI] = −0.04 [−0.30, 0.17], P = 0.103) (Fig. 6, Table 3). However, where PM-CS was applied, mosquito densities were dramatically reduced during the same period of 3 months immediately after spraying (IPE [95 % CI] = 0.93 [0.87, 0.97], P < 0.001). Between the fourth and the sixth month after spraying with DM-WG, there was an apparent, but presumably spurious, 3-fold increase in *An. funestus* densities while LC-CS, PM-EC and PM-CS achieved 5-, 3- and 71-fold reductions, respectively (Table 3). However, from the seventh to 12th months after spraying, DM-WG and PM-EC had no obvious effect on the *An. funestus* densities, while insufficient data were available to examine the incremental impact of LC-CS or PM-CS.

**Table 3:** Association of *Anopheles funestus* densities with different IRS insecticides supplementing LLINs upon months before, during and when not spraying

Indoor residual spraying insecticide treatment regimen	Absolute numbers caught	Mean catches^a^	Relative biting rates of *An. funestus*	
		[95 % Confidence interval (CI)]	(RR)^a^ [95 % CI]	P value
Deltamethrin WG)				
1–3 months since last spray	73	0.112 [0.641, 0.371]	0.99 [0.63, 1.56]	0.897
4–6 months since last spray	1229	0.641 [0.371, 1.109]	3.98 [3.15, 5.04]	<0.001
7–12 months since last spray	134	0.111 [0.062, 0.199]	0.86 [0.64, 1.17]	0.067
>12 months since last spray or never	1186	0.189 [0.113, 0.317]	1 [NA]^b^	NA^b^
Lambdacyhalothrin CS				
1–3 months since last spray	20	0.191 [0.090, 0.405]	1.03 [0.56, 1.88]	0.805
4–6 months since last spray	6	0.055 [0.022, 0.141]	0.17 [0.08, 0.39]	<0.001
7–12 months since last spray	0	NE^c^	NE^c^	0.972
>12 months since last spray or never	182	0.198 [0.121]	1 [NA]^b^	NA^b^
Pirimiphosmethyl EC				
1–3 months since last spray	478	0.234 [0.131, 0.417]	1.04 [0.83, 1.30]	0.786
4–6 months since last spray	346	0.055 [0.030, 0.098]	0.25 [0.20, 0.33]	<0.001
7–12 months since last spray	160	0.159 [0.086, 0.293]	0.69 [0.50, 0.95]	0.151
>12 months since last spray or never	2823	0.234 [0.131, 0.417]	1 [NA]^b^	NA^b^
Pirimiphosmethyl CS				
1–3 months since last spray	14	0.021 [0.009, 0.047]	0.07 [0.04, 0.13]	<0.001
4–6 months since last spray	70	0.004 [0.002, 0.008]	0.02 [0.01, 0.02]	<0.001
7–12 months since last spray	NE^c^	NE^c^	NE^c^	NE^c^
>12 months since last spray or never	2087	0.253 [0.152, 0.422]	1 [NA]^b^	NA

*NA* Not applicable because reference group, *NE* Not estimable because no spraying of this insecticide regimen was conducted early enough to yield impact data beyond 6 months post-spray but before the following spray round so no data are available for estimation

^a^The effect of different IRS treatment regimens on the mean catches of *An. funestus* species where estimated by fitting generalized linear mixed models (GLMMs) with *An. funestus* catches treated as dependent variables. The households where nested within villages which were also nested within the clusters, these together with date were treated as random effects, while the different IRS treatment regimens were categorized as independent variables. A Poisson distribution with no intercept was used to estimate the mean catches while an intercept was included in estimating the RR

### Background observations of insecticide resistance and human exposure profiles for local *Anopheles funestus* populations

**Fig. 7:**
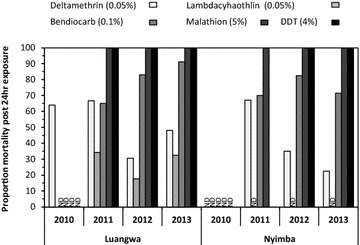
Insecticide resistance profile of *Anopheles funestus* in the study site from 2010 to 2013

From the outset of the study, *An. funestus* exhibited high level of resistance to both pyrethroids against which they were tested, and resistance level generally increased over the course of the study (P < 0.001). Alarming rates of resistance to the carbamate bendiocarb were also observed but these did not increase over the course of the study (P = 0.565). During this same period, there was no evidence of malathion or DDT resistance detected in the mosquito populations (Fig. 7).

**Fig. 8:**
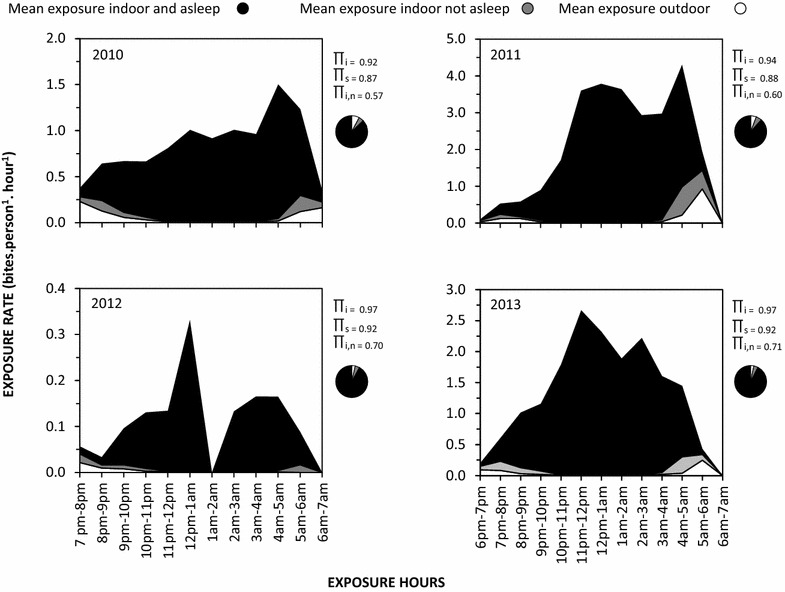
Mean exposure of humans to *Anopheles funestus* bites when they are indoors or outdoors where π_i_ is the average proportion of human exposure to bites of the *Anopheles funestus* which occurs indoors in the absence of any protective measure, π_s_ is the average proportion of human exposure to bites of the *Anopheles funestus* population which occurs indoors when individuals are asleep in the absence of any protective measure, and π_i,n_ is the average proportion of residual human exposure for users of net which occurs indoors, calculated exactly as previously described [39]

Throughout the study period, humans lacking LLINs were exposed to far more bites by *An. funestus* indoors during the late hours of the night up to the early morning hours (Fig. 8), consistent with the known behaviour of *An. funestus* across the continent [42, 49]. The vast majority potential exposure to bites by this dominant vector occurred indoors at times when most individuals are asleep (Fig. 8). Even for those using an LLIN to prevent most indoor transmission, most residual human exposure to *An. funestus* bites and presumably malaria transmission, occurred indoors, increasing gradually from 57 % in 2010 to 71 % by 2013 (Fig. 8).

## Discussion

In this setting of high LLIN unitization (>80 %), even the modest (0–100 %, mean = 29.4 %) coverage achieved with supplementary IRS conferred an incremental protection against malaria parasite infection through reduced vector population density, human exposure to bites and, presumably, to sporozoite inoculations. Overall supplementing of LLINs with IRS using PM-CS gave the greatest apparent protection against malaria risk, which lasted for a full 6 months, while IRS with PM-EC conferred less dramatic protection that was comparable with pyrethroids but apparently lasted for one full year. Neither of the two pyrethroid formulations exhibited any incremental protective effect for more than 3 months, but it is notable that LC-CS conferred an apparently greater protective effect than DM-WG. These observations that quasi-randomly assigned IRS treatments conferred additional protection when provided as a supplement to LLIN utilization, are consistent with a variety of other observational studies [17, 50], as well as more recent randomized controlled studies [51]. The high level of incremental impacts observed, despite sometimes mediocre coverage with IRS, are actually consistent with the predictions of process-explicit models used to support the policy switch to universal coverage for both LLINs and IRS [52], especially for a very anthropophagic mosquito such as *An. funestus* [2], which is even more anthropophagic than the *An. gambiae* species [53] used as an example mosquito in that simulation paper.

The high protective effect of PM-CS is also evident in the low densities of *An. funestus* caught in that group. The modest and short-lived protective effect of the two pyrethroid formulations, DM-WG and LC-CS, most probably a result of the emergence of resistance to pyrethroids in the *An. funestus* population present in this study area, consistent with evidence from Benin in west Africa that the protective effect of these insecticide formulations can be dramatically reduced to as little as a month by physiological resistance, even where these specific formulations have a residual activity against susceptible, insectary-reared, mosquitoes for up to 6 months [33]. While this rapid loss of incremental protection towards malaria elimination with pyrethroid-based supplementary IRS is of obvious and very direct concern [54–56], the encouraging results obtained with IRS using PM, the CS formulation in particular, provide further evidence that pyrethroid resistance may be mitigated and managed in areas of high LLIN coverage using IRS [20, 51], or alternatively impregnating wall linings [57, 58] with non-pyrethroids selected on the basis of standard WHO susceptibility assays. These observations are therefore consistent with similar recent reports from several distinct settings across Africa [51, 59–61] and can be readily rationalized on the basis of the combined observations of strong resistance to pyrethroids, complete susceptibility to organophosphates, and strong tendency to feed and presumably rest indoors among the local *An. funestus* population.

It was expected that PM-CS would be the most persistent because this micro-encapsulated formulation is known to confer residual longevity for 6 months [33, 40] as confirmed here. However, it was surprising that PM-EC had the longest longevity on these surfaces, apparently lasting 12 months after spraying, contrary to other studies suggesting that PM-EC is ineffective on mud surfaces [40] and WHO estimates of a residual effect of only 3 months [62] but is consistent with one other recent study [63]. While it is possible to speculate that the persistence of PM-EC may have resulted from an initial absorption into the porous mud walls in most of the houses in the study areas, followed by slow subsequent release, it is also possible that this is simply the result of a spurious model fit to data from such a limited number of treated clusters with considerable intercluster variation in malaria risk level and seasonality, as presumably occurred for DM-WG, which is highly unlikely to have really increased malaria transmission (Fig. 3, Table 2) or vector density (Fig. 6, Table 3). The observation that impact of both PM formulations and LC-CS upon vector density was greatest between 4 and 6 months after spraying suggests that maximum impact upon the vector population required sustained impact upon several generations of mosquitoes, well into the peak rainy season when they would be expected to grow exponentially and improve in reproductive fitness as the availability of larval habitat rapidly increases [64, 65].

Of course, there are several substantive limitations to this study. While the community-based nature of both the parasitological and entomological surveys, with only modest supervision and quality assurance, does leave some uncertainties about the data quality, recent detailed analyses of these primary [36, 46] and secondary outcomes [34] provide reassuring confirmation of their epidemiological relevance and discriminative power. An additional limitation lies in the lack of a comprehensive quality assurance system for the RDTs results, comparing them with better-established tests, such as microscopy, or more sensitive diagnostic tests, such as polymerase chain reaction. In spite of the known limitations in the sensitivity of RDTs [66], it is encouraging that this specific test kit product, applied in exactly the manner described here, proved a robust means of monitoring infection and disease burden [36, 46]. While this study did not explicitly or comprehensively track the distinct costs of IRS and LLINs, these costs may be assumed to be incurred largely independently of each other because of their distinct delivery methods, and have already been evaluated in detail across a variety of settings by other authors [12, 26, 67, 68]. However, the most obvious limitation of this study is that it was not conducted as a rigorous randomized control trial and that deviations from the original randomization plan resulted in only a quasi-randomized design in practice, with known selection biases. This was also coupled with a lack of a statically estimated sample size. An additional considerable limitation arising from dependency on delivery of supplementary IRS through routine programmatic implementation mechanisms was the lack of consistent availability of a single, optimal formulation of a single pyrethroid or a single formulation of PM, so the study was unfortunately fragmented into more treatment arms with smaller numbers of assigned clusters per spray round than originally planned. Also, delays and limitations in the availability of PM formulations in the final year of the study resulted in a mismatch in the timing of application of PM-CS in Nyimba (November 2012), PM-EC and LC-CS (both February 2013).

So, in summary this study was not fully randomized because the implementation contractors did not fully adhere to the study design stipulated to them by the NMCC. This study may therefore be described as a quasi-randomized experimental evaluation to generate plausible evidence that the IRS treatments provide effective incremental impact beyond that already provided by high coverage with LLINs under near-programmatic conditions. The biggest inherent limitation of observational studies is their vulnerability to selection bias and confounding [69, 70]. The study largely adhered to its original randomization plan, and some of the most confounding variables were taken into consideration during planning and implementation (stratification of clusters into those that had previously been sprayed with deltamethrin and those that had not) and analysis (additional variables in regression models) phases. However, the deviations from the randomization plan by the implementing agencies were specifically necessitated by product stock availability and motivated by efforts to target IRS to areas where they felt it was needed most, so treatment allocation was clearly systematically biased a priori in these cases. It is therefore prudent to interpret the level of evidence generated conservatively, and to classify this study as being essentially observational in nature. To generate probable evidence of efficacy under more precisely controlled (if somewhat less programmatically relevant [71, 72]) conditions would have entailed a rigorous, fully-randomized trial with a registered protocol including sample size estimates, data quality assurance and oversight committees [71–74].

While the shortcomings of this study must be accepted, there is no obvious specific reason to suggest that they are inaccurate, and they do contribute to a relatively limited evidence base regarding the incremental impact of IRS formulations as supplements to LLINs [21]. Despite these study design limitations, the evidence generated remains useful for guiding programmatic selection of IRS treatments. Perhaps just as important, it represents the first effort of the NMCC itself, rather than its specialist research and academic partner institutions, to conduct a cluster-randomized experimental evaluation of malaria transmission control measures. It represents, therefore, an invaluable experience through which the capacity of the NMCC has grown and can hopefully build upon.

## Conclusion

Despite these study limitations, the results presented here do provide substantial evidence that: (1) supplementing pyrethroid-based LLINs with pyrethroid-based IRS confers some, albeit short-lived, incremental protection against malaria infection relative to LLINs alone; and, (2) replacing pyrethroids with an alternative insecticide class, in this case a long-lasting CS formulation of the organophosphate PM, as the active ingredient for supplementary IRS confers considerably enhanced protection, relative to IRS with pyrethroids. Supplementing LLINs with IRS using non-pyrethroids therefore appears to be efficacious for mitigating the immediate epidemiological consequences of vector population resistance to pyrethroids, and the observed impact on *An. funestus* densities suggest it may also be a valuable option for managing such resistance traits, ideally by using mosaics, rotations or combinations of complementary active ingredients [28]. Of course the primary limitation to the realization of such insecticide resistance management and mitigation plans in practice are: (1) the availability of more efficacious, affordable and diverse insecticide formulations [75]; (2) increased financing for malaria vector control generally [76]; and, (3) more cost-effective methods for targeting insecticides to vector populations so that both the biological resource coverage [77, 78] and mortality rates arising from exposure to their active ingredients are maximized [79–83].

## Additional file

10.1186/s12936-016-1143-7 Indicating IRS coverage, LLIN utilization and diagnostic positivity 1 to 6 months post IRS implementation by cluster and sporozoite prevalence, diagnostic positivity with or without IRS treatment and corresponding indicative start point of IRS treatment.

## Abbreviations

AL: artemether lumefantrine
EIR: entomological inoculation rate
CB: community based
CHW: community health worker
IRS: indoor residual spraying
LLINs: long-lasting insecticidal nets
RDT: rapid diagnostic tests
GLMM: generalized linear mixed model
NMCC: National Malaria Control Centre
DMO: District Medical Office
LT: light trap
ITT: Ifakara tent trap
PCR: polymerase chain reaction
HLC: human landing catches
IPE: incremental protective effect
WG: wetable granules
CS: capsule suspension
EC: emulsifiable concentrate
IPT: intermittent presumptive therapy
DP: diagnostic positivity
OR: odds ratios
RR: relative risk
PM: pirimiphos methyl
