# Supplementary material for: Incremental impact upon malaria transmission of supplementing pyrethroid-impregnated long-lasting insecticidal nets with indoor residual spraying using pyrethroids or the organophosphate, pirimiphos methyl
Source: Malar J. 2016 Feb 18;15:100. doi: 10.1186/s12936-016-1143-7 (PMC4758014; doi:10.1186/s12936-016-1143-7)
Supplement: Supplementary file 1 — 10.1186/s12936-016-1143-7 Indicating IRS coverage, LLIN utilization and diagnostic positivity 1 to 6 months post IRS implementation by cluster and sporozoite prevalence, diagnostic positivity with or without IRS treatment and corresponding indicative start point of IRS treatment. [file 12936_2016_1143_MOESM1_ESM.pdf]

Additional file:

IRS coverage, LLIN utilization and Diagnostic Positivity 1 to 6 months post IRS implementation by cluster.

Period	Cluster	IRS Status	IRS Coverage % (n/N)	LLINs use % (n/N)	Diagnostic positivity % (n/N)
**October 2010 to March 2011**	1	None	0.0 (0/1577)	65.1 (1025/1575)	24.7 (372/1508)
	2	None	0.0 (0/827)	98.0 (2485/2537)	20.9 (559/2676)
	3	None	0.0 (0/2182)	49.0 (1492/3046)	26.9 (809/3006)
	4	Deltamethrin WG	72.0 (1920/2667)	82.3 (2194/2667)	33.2 (825/2489)
	5	Deltamethrin WG	92.4 (1362/1474)	95.8 (1412/1474)	27.5 (396/1439)
	6	Deltamethrin WG	95.3 (2750/2886)	95.4 (2744/2876)	11.9 (338/2845)
	7	Deltamethrin WG	98.5 (2358/2395)	99.7 (2467/2474)	6.0 (144/2415)
	8	None	0.0 (0/424)	6.6 (28/426)	55.7 (202/363)
	9	None	0.0 (0/7)	36.4 (4/11)	36.4 (4/11)
	10	None	0.0 (0/343)	39.7 (136/343)	50.7 (172/339)
	11	None	0.0 (0/134)	32.1 (43/134)	51.3 (60/117)
	12	None	0.0 (0/49)	79.6 (39/49)	61.9 (26/42)
	13	None	0.0 (0/221)	30.8 (68/221)	60.0 (120/200)
	14	None	0.0 (0/77)	41.6 (32/77)	52.4 (33/63)
**October 2011 to March 2012**	1	None	0.0 (0/1105)	87.4 (1063/1217)	9.5 (95/998)
	2	Pirimiphosmethyl EC	10.3 (245/2380)	75.8 (2235/2948)	8.5 (280/3292)
	3	None	0.0 (0/2593)	33.1 (1253/3790)	10.8 (436/4033)
	4	Pirimiphosmethyl EC	57.9 (2194/3788)	92.9 (3541/3811)	5.9 (217/3708)
	5	Pirimiphosmethyl EC	57.6 (1952/3392)	97.7 (3388/3469)	18.2 (624/3436)
	6	Lambdacyhalothrin CS	80.7 (1128/1398)	80.2 (1139/1421)	5.2 (76/1457)
	7	Lambdacyhalothrin CS	81.8 (2317/2833)	99.9 (3107/3109)	4.2 (130/3111)
	8	None	0.0 (0/3918)	75.4 (2952/2917)	29.9 (974/3261)
	9	Pirimiphosmethyl EC	33.5 (541/1613)	51.95 (838/1613)	46.6 (684/1467)
	10	None	0.0 (0/1466)	55.4 (812/1466)	35.4 (444/1254)
	11	Pirimiphosmethyl EC	1.8 (74/4117)	60.1 (2474/4117)	30.2 (941/3112)
	12	None	0.0 (0/1546)	59.7 (925/1549)	33.9 (514/1517)
	13	Pirimiphosmethyl EC	16.8 (407/2422)	45.9 (1111/2422)	27 (5.033/1974)
	14	None	0.0 (0/1964)	52.1 (1023/1964)	41.3 (786/1904)
**October 2012 to March 2013**	1	None	0.0 (0/464)	100.0 (464/464)	14.4 (150/1039)
	2	None	0.0 (0/1076)	99.8 (1074/1076)	11.9 (126/1061)
	3	None	0.0 (0/1915)	99.1 (1897/1915)	14.1 (282/2004)
	4	Pirimiphosmethyl EC	100 (1170/1170)	100.0 (2632/2632)	10.8 (314/2908)
	5	Pirimiphosmethyl EC	100 (1387/1387)	99.7 (1725/1730)	27.3 (456/1673)
	6	Lambdacyhalothrin	100 (348/348)	100.0 (1386/1386)	3.8 (57/1505)

	CS			
7	Lambdacyhalothrin CS	88.8 (645/726)	100.0 (1123/1123)	2.99 (33/1105)
8	Pirimiphosmethyl CS	0.7 (18/2439)	28.5(696/2439)	9.0 (209/2321)
9	Pirimiphosmethyl CS	73.7 (1386/1881)	48.5 (913/1881)	23.5 (366/1561)
10	Pirimiphosmethyl CS	0.7 (10/1379)	85.1 (1174/1379)	27.1 (363/1341)
11	None	0.0 (0/3440)	16.5 (566/3440)	11.9 (300/2531)
12	Pirimiphosmethyl CS	30.3 (346/1143)	82.2 (940/1143)	21.7 (246/1132)
13	None	0.0 (0/2433)	46.4 (1128/2433)	30.6 (666/2180)
14	Pirimiphosmethyl CS	40.4 (574/1421)	38.1 (541/1421)	16.99 (221/1301)

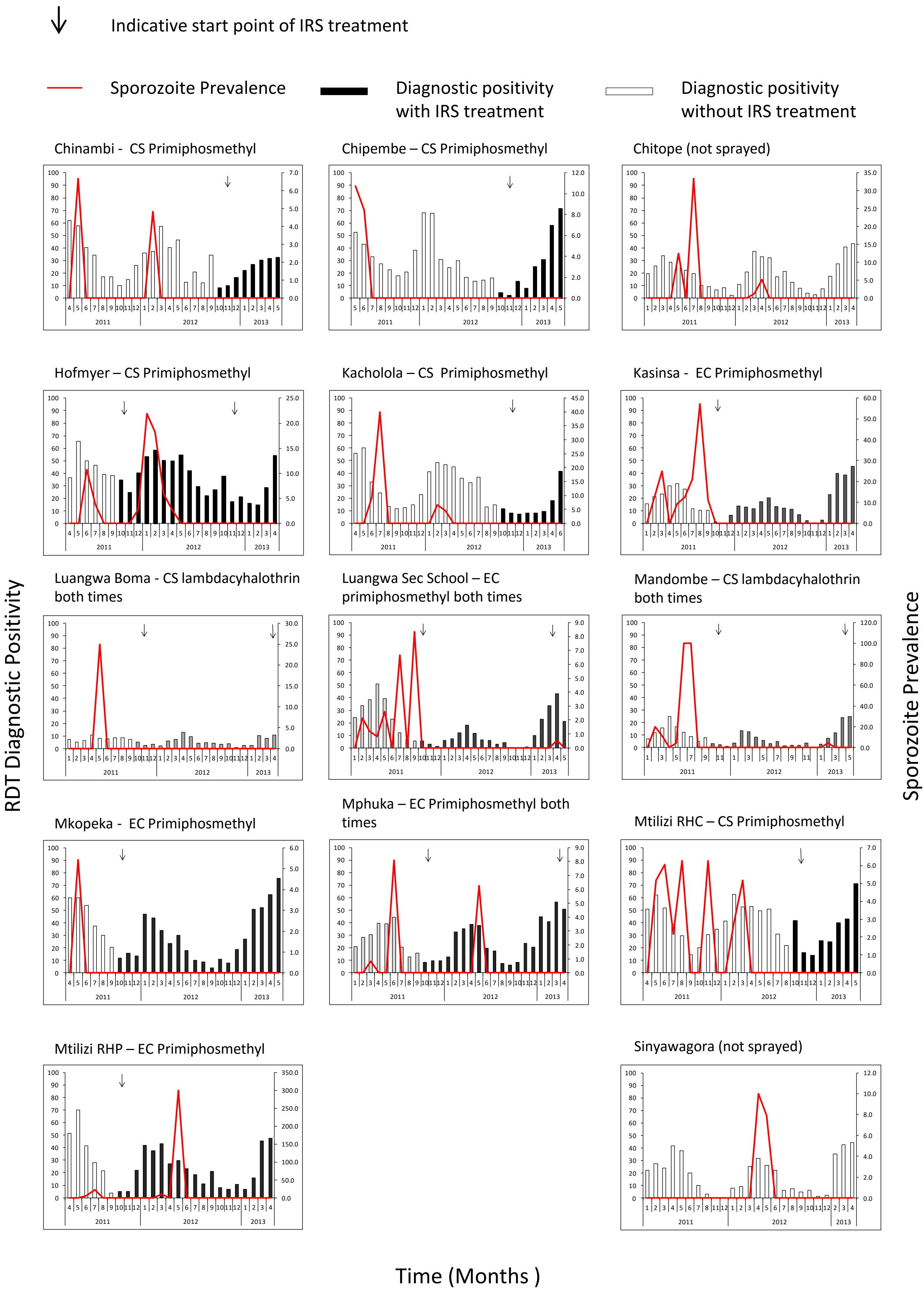

Indicative start point of IRS treatment
Sporozoite Prevalence
Diagnostic positivity with IRS treatment
Diagnostic positivity without IRS treatment
Chinambi - CS Primiphosmethyl
Chipembe – CS Primiphosmethyl
Chitope (not sprayed)
Hofmyer – CS Primiphosmethyl
Kacholola – CS Primiphosmethyl
Kasinsa - EC Primiphosmethyl
Luangwa Boma - CS lambdacyhalothrin both times
Luangwa Sec School – EC primiphosmethyl both times
Mandombe – CS lambdacyhalothrin both times
Mkopeka - EC Primiphosmethyl
Mphuka – EC Primiphosmethyl both times
Mtilizi RHC – CS Primiphosmethyl
Mtilizi RHP – EC Primiphosmethyl
Sinyawagora (not sprayed)
RDT Diagnostic Positivity
Sporozoite Prevalence
2011
2012
2013
Time (Months)
